# Hospital Problems in India

**Published:** 1920-07-31

**Authors:** 


### HOSPITAL PROBLEMS IN INDIA.

### HEALTH AND MATERNITY TRAINING.

From a Correspondent.

The following are the particulars of the training to be carried on in the Delhi Institute by the Association for the Provision of Health and Maternity Supervisors, of which Lady Chelmsford is President.

There are two standards of training :

(*a*) Health and Maternity Supervisors, with one year's training, equal to that given in England under the regulations of the Board of Education.

(*b*) Health and Maternity Visitors with six months' training.

The duties of (*a*) will be to supervise and improve the work of midwives and *dais*—*i.e.*, native midwives—as well as to visit Indian mothers in their homes as stated under (*b*). The duties of (*b*) to visit homes, especially where there are young infants, and instruct mothers in child welfare and sanitation. Quarters have been secured in Nicholson Road, Delhi. Pupils will live under the supervision of the lady health visitors with the Delhi Municipality. Each candidate should be over twenty-one and hold certificates showing she has had a satisfactory educational standard.

For (*a*) this standard should be matriculation of an Indian University or its equivalent. For (*b*) the preliminary education should be the seventh standard. Candidates knowledge of English must be good, but a vernacular tutor will be employed to help pupils. Each candidate should have a training in nursing and midwifery at an approved hospital. She must also produce satisfactory certificates of health and moral character. The course of training for (*a*) will commence on October 1, and for (*b*) on January 5 each year. During the first month pupils will be on probation. Instruction will be as follows :

(*a*) Lectures on physiology, home hygiene, and general sanitation, tropical diseases, tuberculosis, diseases of infancy and childhood, pre-maternity and infant welfare, bookkeeping as carried on in infant-welfare centres; elementary economics, sick-room cookery.

(*b*) Practical demonstrations on general sanitation and home hygiene; visiting and supervising of maternity cases; first-aid and home nursing (especially with regard to children's ailments).

(*c*) Lantern lectures and talks to mothers; conducting classes for *dais* (Indian midwives).

A qualifying examination will be held in the latter half of July. Successful pupils receive diplomas as (*a*) trained health and maternity supervisors, and (*b*) trained health and maternity visitors.

The Examining Board will include the Sanitary Commissioner with the Government of India, the Sanitary Commissioner with the Government of the Punjab, and the Health Officer Imperial Delhi.

